# Supplementary material for: Predict or classify: The deceptive role of time-locking in brain signal classification
Source: Sci Rep. 2016 Jun 20;6:28236. doi: 10.1038/srep28236 (PMC4913298; doi:10.1038/srep28236)
Supplement: Supplementary Information [file srep28236-s1.pdf]

Supplementary note

Predict or classify: The deceptive role of time-locking in brain signal classification

Marco Rusconi, Angelo Valleriani

S1 Support-vector classification

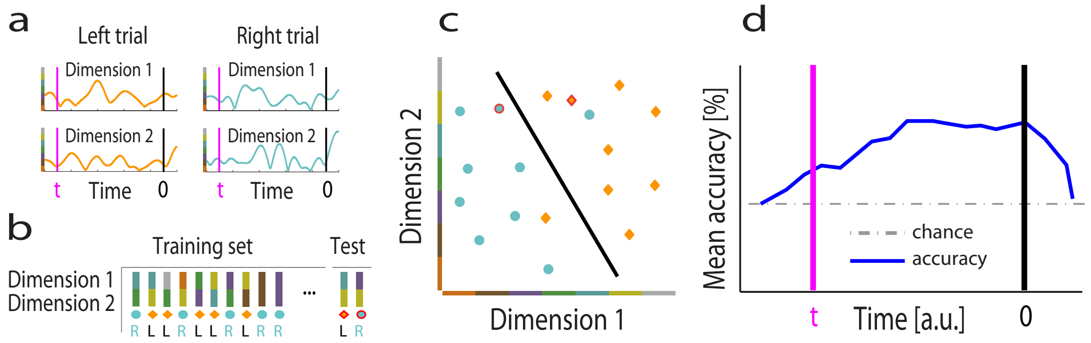

Supplementary Figure 1: **Multivariate classification with cross validation of left/right button press trials.** (**a**) In the first step, the trials are time-locked to the time of button press ($t = 0$). The value at each time t of the multidimensional imaging signal define the multivariate pattern for each of the $M + M$ trials. To exemplify, we show here one "left" and one "right" trial with a bivariate signal. (**b**) The values at t form the pattern used during the classification. All pairs of "left" and "right" trials but one are used to train a linear classifier to learn to distinguish the typical "left" and "right" trials. The remaining pair is then used to test the learned model. This procedure is repeated until each pair of trials is used for testing. (**c**) The linear classifier finds a classification hyperplane using the trial set. For each of the test sets it verifies if both are on the correct side (100% accuracy) only one is correct (50% accuracy) or none is correct (0% accuracy). (**d**) At each t the average accuracy across all M tested pairs is computed. By repeating the procedure at each time point, one maps the time t versus the mean accuracy with which the button press was decoded. When several participants are involved, this procedure is repeated for each participant and the outcome is then averaged.

S2 The effect of time-locking with a complex network

In this section we discuss the effect that time-locking has on data generated with an example of a network with a more complex topology than a linear network and a complex graph. To obtain this network we introduced extra links and loops between the nodes of the linear network. The inset of Supplementary Figure 2 shows the topology that we used. After generating the data with this new network, we repeated the analysis presented in the main text. We therefore computed both the decoding accuracy, time-locked to $t = 0$, and the corresponding mutual information.

In Supplementary Figure 2 we show the time course of the decoding accuracy for the complex network and the benchmark linear network. Introducing links between the nodes allowed a more complex topology that resulted in a faster decay of the decoding accuracy while going backwards from $t = 0$. The decay rate is however intermediate between linear and complete graph.

In Supplementary Figure 3 we show the time course of the time-locked mutual information for the case of the complex network. Also in this case, decoding accuracy and mutual information have a qualitatively similar behavior. The unconstrained mutual information is however equal zero also in this case. This latter analysis extends to the dynamics of a complex network the results presented in the main text.

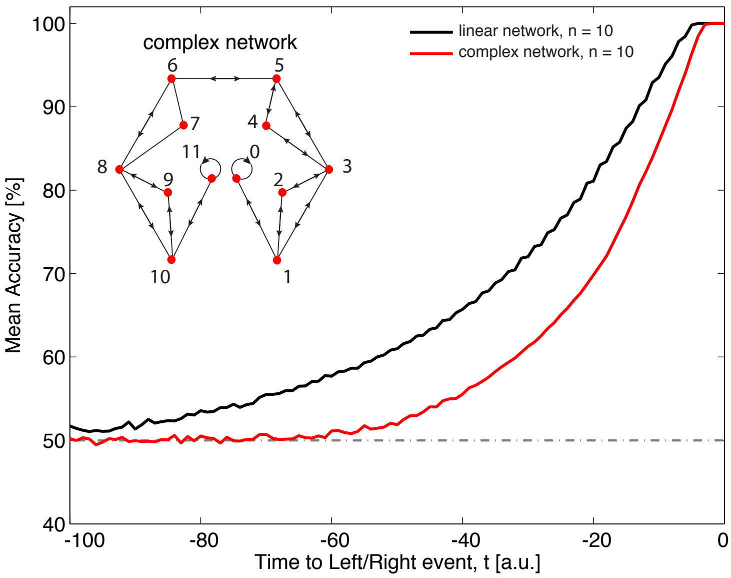

Supplementary Figure 2: **Decoding accuracy.** The main figure shows the time course of the decoding accuracy for the linear (black line) and a complex network (red line) both with $n = 10$ states. The inset shows the topology of the latter. Compared with the complete graph, in the complex network the topology is sparse but is more connected than the linear chain. The presence of few additional links results in a faster decaying to chance level (50%) of the decoding accuracy moving backward from $t = 0$.

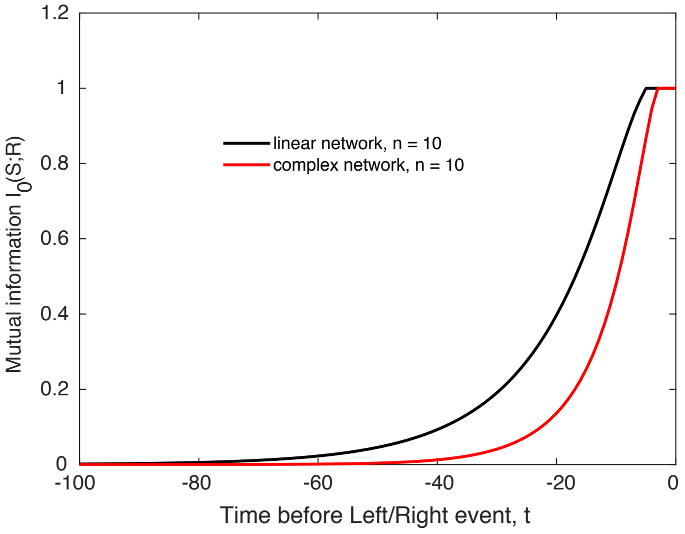

Supplementary Figure 3: **Mutual information analysis.** The plot shows the time-locked mutual information $I_0(S; R)$ for the linear (black) and a complex (red) network. This last one is the same of Supplementary figure 2. Consistently with the results reported in the main text, the decoding accuracy and the time-locked mutual information show a qualitatively similar behavior. Going backward from $t = 0$ they both decay and this decays is faster for a sparse network than for a linear graph.
